# Selective degradation of AR-V7 to overcome castration resistance of prostate cancer

**DOI:** 10.1038/s41419-021-04162-0

**Published:** 2021-09-21

**Authors:** Yuan Liu, Cuifu Yu, Zhenlong Shao, Xiaohong Xia, Tumei Hu, Weiyao Kong, Xiaoyue He, Wenshuang Sun, Yuanfei Deng, Yuning Liao, Hongbiao Huang

**Affiliations:** 1 Affiliated Cancer Hospital & Institute of Guangzhou Medical University, 510095 Guangzhou, Guangdong China; 2 Guangzhou Municipal and Guangdong Provincial Key Laboratory of Protein Modification and Degradation, School of Basic Medical Sciences, Guangzhou Medical University, 511436 Guangzhou, Guangdong China; 3 Department of Pathology, First People’s Hospital of Foshan, 528000 Foshan, Guangdong China

**Keywords:** Drug development, Translational research

## Abstract

Androgen receptor splice variant 7 (AR-V7), a form of ligand-independent and constitutively activating variant of androgen receptor (AR), is considered as the key driver to initiate castration-resistant prostate cancer (CRPC). Because AR-V7 lacks ligand-binding domain, the AR-targeted therapies that aim to inactivate AR signaling through disrupting the interaction between AR and androgen are limited in CRPC. Thus, the emergence of AR-V7 has become the greatest challenge for treating CRPC. Targeting protein degradation is a recently proposed novel avenue for cancer treatment. Our previous studies have been shown that the oncoprotein AR-V7 is a substrate of the proteasome. Identifying novel drugs that can trigger the degradation of AR-V7 is therefore critical to cure CRPC. Here we show that nobiletin, a polymethoxylated flavonoid derived from the peel of *Citrus* fruits, exerts a potent anticancer activity via inducing G0/G1 phase arrest and enhancing the sensitivity of cells to enzalutamide in AR-V7 positive PC cells. Mechanically, we unravel that nobiletin selectively induces proteasomal degradation of AR-V7 (but not AR). This effect relies on its selective inhibition of the interactions between AR-V7 and two deubiquitinases USP14 and USP22. These findings not only enrich our understanding on the mechanism of AR-V7 degradation, but also provide an efficient and druggable target for overcoming CRPC through interfering the stability of AR-V7 mediated by the interaction between AR-V7 and deubiquitinase.

## Introduction

Prostate cancer (PC) is the second most common male cancer with high lethality worldwide. In 48 countries, mainly in sub-Saharan regions, the Caribbean, Central and South America, and Northern Europe, it is the leading cause of cancer deaths among the male [1, 2]. Androgen receptor (AR), a ligand-dependent transcription factor appertaining to the nuclear receptor family, is a core driver of the development and progression of PC as well as the key treatment target [3]. Androgen deprivation therapy (ADT) by surgical or chemical castration to decrease circulating testosterone levels and to inhibit cellular AR signaling pathway, has been successfully used in advanced prostate cancer. Although ADT displayed great effect in patients with hormone-sensitive prostate cancer at first, most cases tend to gradually develop resistance to this therapy. PC at this stage is therefore called castration-resistant prostate cancer (CRPC) and is considered incurable [4, 5].

Reactivation of AR signaling is frequently observed in CRPC. Despite a variety of schemes, including repression of androgen synthesis, blockade of AR nuclear translocation, and inhibition of AR function, etc., are used to inhibit the AR signaling pathway, the reactivation of this signaling served for the growth of CRPC cells can still be detected [4–6]. In terms of mechanism, the reasons mainly comprise AR mutation [7], AR amplification [8], AR genomic rearrangement [9], and the expression of AR splice variants (AR-Vs) [10, 11]. Among the numerous AR-Vs that have been described [12], AR-V7 is the most attractive one with the highest frequency of detection and important clinical relevance [5, 10, 11]. Numerous studies have pointed out that AR-V7, a form of AR-Vs lacks the ligand-binding domain (LBD), exhibits a ligand-independent and constitutively activated manner [5, 10, 13], suggesting that AR-V7 can enter the nucleus to produce AR-like functions but does not rely on androgen stimulation. Additionally, AR-V7 can also combine with the full-length AR (AR-FL) and subsequently enter the nucleus together, thereby leading to the reactivation of the AR signaling [14]. Although the expression of AR-V7 has been proposed as the main driver of CRPC progression, there is currently no effective approach to counteract its activity.

Nobiletin (3′,4′,5,6,7,8-hexamethoxyflavone) is a polymethoxylated flavonoid extensively derived from the peel of *Citrus* fruits [15]. Multiple biological activities of nobiletin and its derivatives have been reported, including anti-insulin resistance [16], antioxidant [17], anti-inflammatory [17, 18], anticancer [19, 20], cardiovascular protection [21], and neuro-protection [22], etc. However, the activity of nobiletin on CRPC remains to be elucidated. This research aims to explore the anti-CRPC effect of nobiletin and clarify its underlying mechanism. We showed that nobiletin is able to suppress the growth of CRPC. Additionally, nobiletin can also restore the sensitivity of CRPC cells to enzalutamide, a potent AR antagonist. Further studies on molecular biology revealed that nobiletin selectively induced AR-V7 degradation by inhibiting the interaction of AR-V7 with deubiquitylating enzymes (DUBs), including the ubiquitin-specific protease 14 (USP14) and 22 (USP22). Our research will deepen the comprehension of the regulatory mechanism of AR-V7, and will providea novel theoretical basis for the clinical treatment of CRPC by using a natural product.

## Materials and methods

### Cell culture and reagents

Based on our previous reports [23, 24], WPMY-1 (the human prostate epithelial cell line) cells were cultured in DMEM (Gibco, Invitrogen, Paisley, UK) with 10% fetal bovine serum (FBS). 22Rv1 and C4-2 cells were cultured in RPMI-1640 (Gibco, Invitrogen, Paisley, UK) with 10% FBS. PC3 and DU145 cells were cultured in DMEM/F12 (Gibco, Invitrogen, Paisley, UK) with 10% FBS. The above cell lines were cultured in an incubator at 37 °C with 5% CO_2_, which were purchased from the American Type Culture Collection (Manassas, VA, USA), and authenticated by short tandem repeat (STR), morphology, cell viability, and routine mycoplasma assays. Antibodies and chemicals were shown in Tables S1 and S2.

### Cell proliferation assays

Cell proliferation assays were performed as previously reported [23, 24], including cell viability, colony formation, and EdU staining. The cell counting kit-8 (CCK8) assay (CK04) (Dojindo Molecular Technologies, Japan) was used to detect cell viability. Simply, the cells were seeded in 100 μl of complete medium at a concentration of 2000 cells per well in a 96-well plate for 24 h, and then treated according to the conditions indicated in figure legends. Then according to the CCK8 manufacturer’s instructions, a microplate reader (Sunrise reader, Tecan, Mannedorf, Switzerland) was used for the absorbance detection at a wavelength of 450 nm from three independent experiments.

The EdU assay(RiboBio, Guangzhou, China) was used to detect DNA replication in cells. The cells were planted on a detachable chamber slide for 24 h, then treated with different concentrations of nobiletin indicated as the figure, and then stained according to the instructions of the EdU kit. After mounting the slides, the images of three independent experiments were randomly captured by Olympus microscope for positive rate statistics.

For colony formation assay, the cells were seeded in 35 mm dishes, and treated with the specified dose of nobiletin for 24 h, then washed with PBS and digested with trypsin. Then, the cells were resuspended, seeded on six-well plate and cultured 2 weeks. The cells were fixed with 4% paraformaldehyde for 10 min, then washed with PBS, stained with 1% crystal violet solution for 5 min. Finally, the number of colonies was counted.

### Flow cytometry analysis

Flow cytometry analysis was used to detect the cell cycle distribution and apoptosis. This assay was performed as previously reported [23].

### Real-time quantitative PCR assay

As previously described [25], we extracted total RNAs from 22Rv1 cells treated with nobiletin as the conditions indicated in the figure legend, and measured the RNA purity and concentration at 260:280 nm. After diluting RNAs to the same concentration with RNase-free water, we used PrimeScript™ RT Master Mix (Takara Biotechnology, Dalian, China) to reversely transcribe 1 μg total RNAs and synthesize the first-strand cDNA according to the instructions. Then, real-time PCR was performed to detect β-actin (ACTB), and AR-V7 mRNA levels with TB Green® Premix Ex Taq™ II (Takara Biotechnology, Dalian, China) according to the instructions.Three independent experiments were performed.

Primers were listed as follows: ACTB forward: 5′-TTCTACAATGAGCTGCGTGTG-3′, ACTB reverse: 5′-GGGGTGTTGAAGGTCTCAAA-3′ [26]; AR-V7 forward: 5′-CAGCCTATTGCGAGAGAGCTG-3′, AR-V7 reverse: 5′-GAAAGGATCTTGGGCACTTGC-3′ [27].

### RNA interfering

The RNA interfering assay by siRNA was used to downregulate the expression levels of the targeted gene. Briefly, mix 500 μl RPMI opti-MEM (Gibco, Invitrogen, Paisley, UK), 6 μl lipofectamine RNAiMax (Invitrogen), and 10 μl USP22 siRNAs or control siRNAs were added into the cells that have been preinoculated and cultured in dishes for 24 h with a final concentration of the siRNAs at 30–50 nM. Cells were treated for 48 h for other analysis. The USP14 and USP22 siRNAs were purchased from RiboBio (Guangzhou, China).

The siRNA sequences are listed below: USP14 siRNA-1: 5′-CTGGCATATCGCTTACGTT-3′;USP14 siRNA-2: 5′-TTGCCGAGAAAGGTGAACA-3′;USP22 siRNA-1:5′-GCAGCGAAAAGCTTGGAAA-3′;USP22 siRNA-2:5′-GTACGGAGGCATCTACTGT-3′.

### Plasmid and lentivirus transfection

The plasmids and lentivirus transfection assays were used to upregulate the expression levels of the contained gene. For plasmid transfection, the full length of human USP14 CDS plasmids (CMV-MCS-Flag-SV40-neomycin), the full length of human USP22 CDS plasmids (CMV-MCS-6His-SV40-neomycin), the full length of human AR-V7 CDS plasmids (CMV-MCS-HA-SV40-neomycin), and their control plasmids were constructed and purchased from GeneChem (Shanghai, China). Mix Lipofectamine 3000 (Invitrogen) (2 μl P3000 and 2 μl lipofectamine 3000) and 1 μg plasmids in 500 μl RPMI opti-MEM (Gibco) were added into the cells that have been preinoculated and cultured in dishes for 24 h with a final concentration of the plasmids at 0.75 μg/ml.Cells were treated for 48 h for other analysis.

For lentivirus transfection, lentivirus (Ubi-MCS-HA-SV40-puromycin) containing the full length of human AR-V7 CDS and its control lentivirus were also constructed and purchased from GeneChem (Shanghai, China). After the cells were seeded in dishes or plates and cultured for 24 h, the fresh medium containing 5 μg/ml polybrene(Santa Cruz, CA) and lentivirus at a multiplicity of infection of 10 was used to culture for 48 h. Puromycin at a concentration of 2 μg/ml was used to select and retain the successfully infected cells.

### Immunofluorescence assay

Since the existing antibodies (anti-AR-V7 and anti-USP22) do not support the immunofluorescence assay, we used the tag antibody to bind the exogenous labeled target molecule in the cell, and then used the fluorescent secondary antibody to identify them. This assay was mentioned in a previous study [23]. The image J software was used for picture analysis and data quantification.

### Western blotting and co-IP

Western blotting, a routine assay, was performed as we described before [28]. For Co-IP assay, Dynabeads™ Co-Immunoprecipitation Kit (#14321D) (Carlsbad, CA, USA) was used according to the instructions of the kit. Briefly, after 16–24 h incubation on the rotary mixer, the antibodies were adsorbed on the dynabeads, and then the protein extracts and the antibodies-beads mixture were allowed to react on the rotary mixer for 1–2 h. The protein that was not bound to the antibodies-beads complex was removed by washing, and the protein specifically bound to the antibodies-beads complex was denatured and separated from the magnetic bead by heating and centrifugation, and dissolved in the blue loading buffer. The subsequent western blot analysis was performed.

### Animal models

All animal experiments were approved by institutional animal care and used committees of Guangzhou Medical University and performed as previously described [29]. Male BALB/c Nude mice were purchased from SPF Biotechnology Co., Ltd. (Beijing, China), and raised in the animal center of Guangzhou Medical University. The 22Rv1 cells at a concentration of 2 × 10^6^ cells/100 μl PBS/mice were inoculated subcutaneously on the backflanks of nude mice aged 5–6 weeks. After 1 week, the nude mice with successful xenogeneic tumor transplantation were randomly divided into four groups (eight mice per group), then treated with 40 mg/kg/2d (i.p.) of nobiletin, 25 mg/kg/2d (p.o.) of enzalutamide, combination of nobiletin and enzalutamide, and drug solvent for 25 days. The body weight of nude mice, tumor volume, and weight were measured. Tumor tissues and liver and kidney tissues were taken for further experiments.

### Immunohistochemistry (IHC) and H&E staining assays

Tumor tissues and liver and kidney tissues were fixed with Formalin, embedded in paraffin and sectioned according to standard techniques. For IHC assay, tumor section samples of 22Rv1 were immunohistochemically stained with MaxVision kit (Mixin Biotech) and AR-V7, Ki67 antibodies, according to the kit and antibody instructions. Then, the slides were counterstained with hematoxylin, and the primary antibodies were detected by the DAB method. For H&E staining assay, the paraffin sections of liver and kidney tissue of nude mice were deparaffinized in xylene and washed with ethanol solution of decreasing concentration (95%, 90%, 80%, and 70%) and distilled water, and then stained by Hematoxylin and Eosin Staining Kit (Beyotime, Shanghai, China) according to the kit and antibody instructions.

### Statistical analysis

Experimental data are presented as mean ± S.D. from three independent experiments where applicable. In order to determine statistical probabilities, unpaired Student’s *t-*test or one-way ANOVA is used where appropriate. GraphPad Prism 9 software and SPSS 16.0 were used to statistical analysis. A *P* value <0.05 was considered statistically significant. *P < 0.05, **P < 0.01, ***P < 0.001, ****P < 0.0001.

## Results

### Nobiletin suppresses the proliferation of CRPC cells

**Fig. 1:**
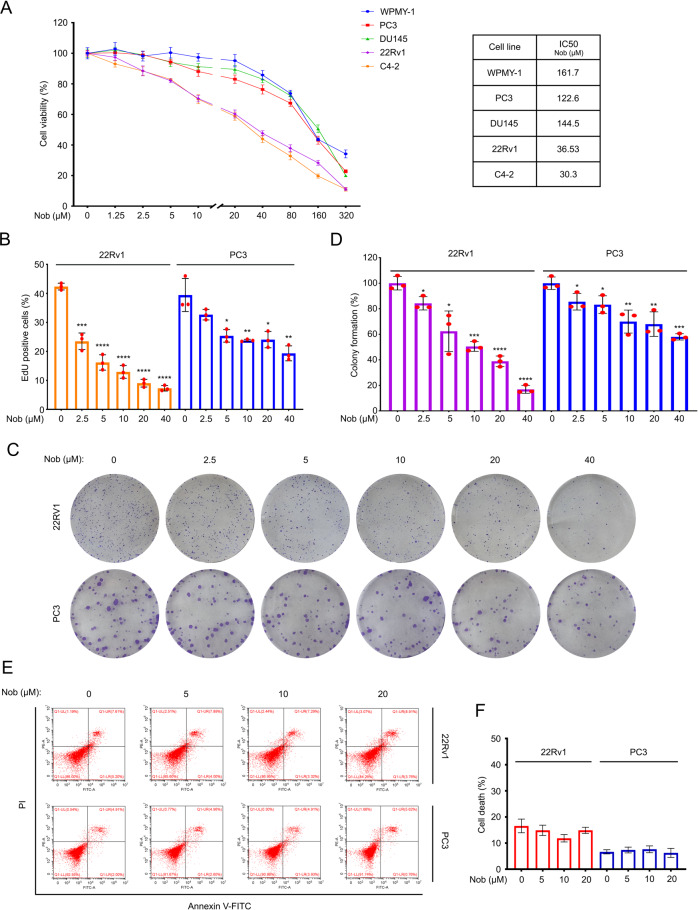
Nobiletin more dramatically suppressed the proliferation of AR/AR-V7-positive prostate cancer cells. **A** Left: CCK8 assays of WPMY-1, PC3, DU145, 22Rv1, and C4-2 cells exposed to nobiletin for 48 h. Mean ± SD (*n* = 3), Nob nobiletin. Right: the IC50 values of nobiletin on different cells. **B** EdU staining assays of 22Rv1 and PC3 cells exposed to nobiletin for 24 h. Quantitative data are shown. Mean ± SD (*n* = 3). **C** Colony formation assays of 22Rv1, and PC3 cells exposed to nobiletin for 2 weeks. **D** Quantification of **C** are shown. Mean ± SD (*n* = 3). **E** Apoptosis analysis of 22Rv1 and PC3 cells treated with nobiletin for 24 h. **F** Quantification of **E** are shown. Mean ± SD (*n* = 3).

To explore the antitumor effect of nobiletin on PC, first of all, cell viability assay was performed in four PC cell lines (PC3, DU145, 22Rv1, and C4-2) and a prostatic stromal myofibroblast cell line WPMY-1. We found that nobiletin significantly reduced the viability of these PC cells, but not the viability of normal prostate cells. Meanwhile, we unexpectedly found that the inhibitory effect of nobiletin in 22Rv1 and C4-2 cells was more obvious than that in PC3 and DU145 cells (Fig. 1A). It is worth noting that according to our previous research [23], 22Rv1 and C4-2 cell lines are AR/AR-V7 positive (especially the 22Rv1 cell line has a higher AR-V7 expression level), but PC3 and DU145 cell lines are AR/AR-V7 negative. The proliferation ability and colony forming ability of the PC cells were determined by using EdU staining assay and colony formation experiment post nobiletin treatment (Fig. 1B–D). The results showed that nobiletin more significantly inhibited the ability of proliferation and colony formation of 22Rv1 than that of PC3. These findings indicate that AR/AR-V7 may be involved in the nobiletin-triggered proliferation suppression.

In addition, this study also ruled out the possibility of nobiletin on inducing apoptosisin the current concentration range and exposure time through Annexin V-FITC/PI staining analyzed by flow cytometry (Fig. 1E, F), indicating that nobiletin-triggered proliferation suppression of PC cells is not associated with apoptosis induction.

### Nobiletin arrests cell cycle in CRPC cells

**Fig. 2:**
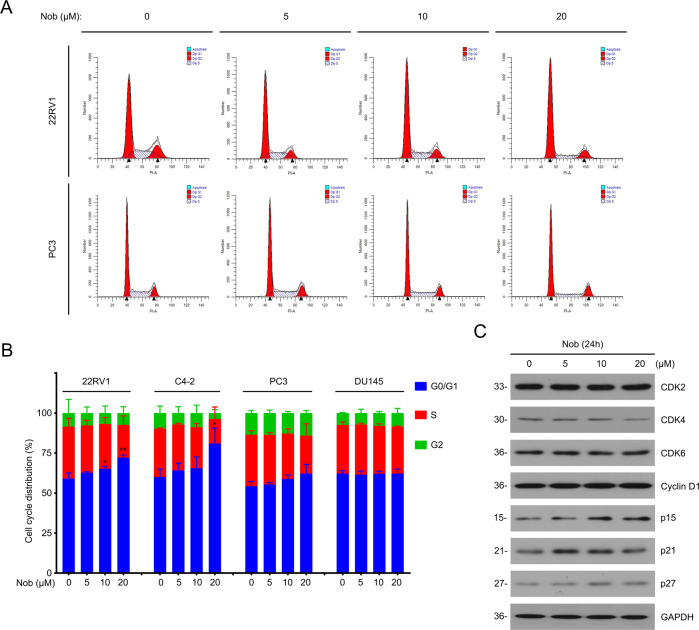
Nobiletin arrested cell cycle in CRPC cells. **A** Cell cycle distributions by flow cytometry analysis of 22Rv1, C4-2, PC3, and DU145 cells treated with nobiletin for 24 h. Here 22Rv1 and PC3 are shown as representative pictures. **B** Quantitative data of **A** are shown from three independent experiments. **C** Western blot analysis of indicated proteins in 22Rv1 cells treated with nobiletin for 24 h.

Next, we further studied the mechanism by which nobiletin inhibits the growth progression of PC cells. Cell cycle checkpoint is a key regulatory factor in the proliferation process of cancer cells.Therefore, we tested whether nobiletin caused changes in cell cycle progression (Fig. 2A, B). The flow cytometry results presented that nobiletin triggered more observable G0/G1 phase arrest in AR/AR-V7-positive cells, which is consistent with the previous proliferation results. At the molecular level, although the protein levels of the sponsors of cell cycle transition from G0/G1 to S phase (including CDK2/4/6 and Cyclin D1) were not altered, expressions of the suppressors (including p15/21/27) were notably upregulated by the treatment of various concentrations of nobiletin (Fig. 2C). It is reasonable to speculate that nobiletin may block the transition from G0/G1 to S phase by increasing the expression levels of p15/21/27. Together, we demonstrate that nobiletin suppresses the proliferative ability of CRPC cells by arresting cell cycle.

### Nobiletin selectively promotes the proteasome-mediated AR-V7 degradation by sponsoring the Lys(K)48-ubiquitinaed form of AR-V7

**Fig. 3:**
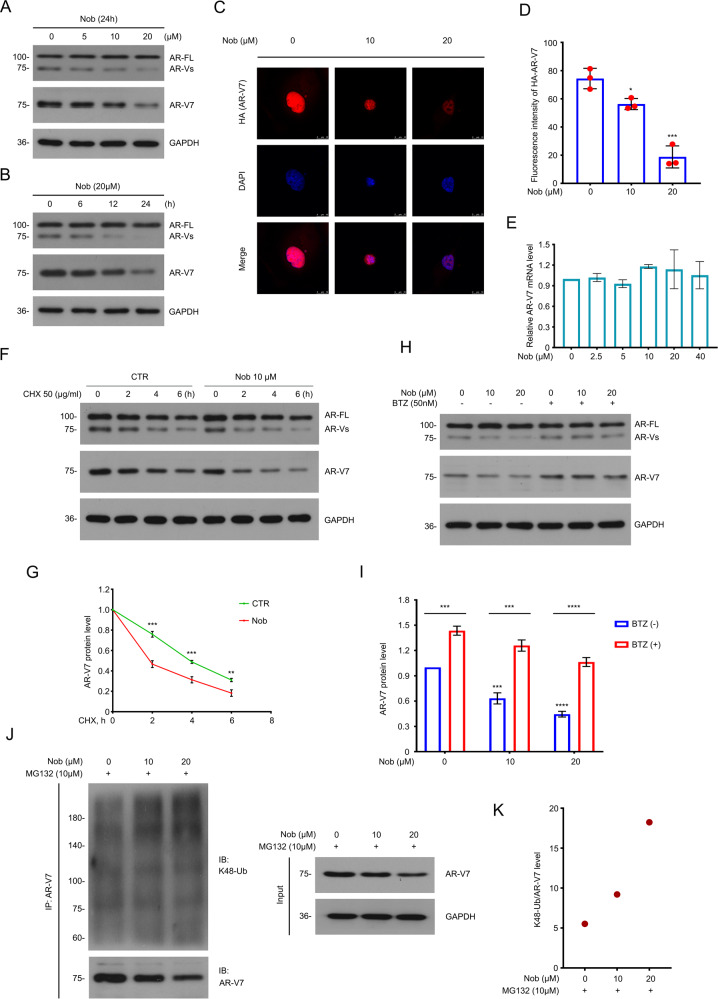
Nobiletin functioned as a selective inducer for degradation of AR-V7. **A** Western blot of AR and AR-V7 in 22Rv1 cells exposed to nobiletin for 24 h.**B** Western blot analysis of AR and AR-V7 in 22Rv1 cells exposed to nobiletin (20 μM) for different lengths of time. **C** Immunofluorescence assay was performed using HA-tag antibody in 22Rv1 cells transfected with HA-AR-V7 plasmids for 48 h and exposed to nobiletin for 24 h. **D** Quantitative data of **C** are shown. Mean ± SD (*n* = 3). **E** Real-time quantitative PCR analysis of AR-V7 in 22Rv1 cells treated with nobiletin for 6 h. **F** Western blot of AR and AR-V7 protein level in 22Rv1 cells treated with nobiletin (10 μM) or DMSO for 12 h, and then exposed to cycloheximide (CHX). **G** Quantitative data of **F** are shown. Mean ± SD (*n* = 3). **H** Western blot analysis of AR and AR-V7 in 22Rv1 cells exposed to nobiletin with or without Bortezomib (BTZ) for 12 h. **I** Quantitative data of **H** are shown. Mean ± SD (*n* = 3). **J** Co-IP assay was performed using AR-V7 antibody and immunoblotted for K48-Ub and AR-V7 in 22Rv1 treated with nobiletin for 12 h, and exposed to MG132(10 μM) for 6 h before harvest. **K** Quantification of K48-ubiquitination levels of AR-V7 for **J**.

As mentioned above, nobiletin showed a certain degree of AR/AR-V7 dependence on suppressing the proliferation of PC cells in vitro.In order to explore the mechanisms, we first selected the 22Rv1 (a classic cell line with high AR and AR-V7 levels) as the experimental object, and then used western blot assay to detect whether nobiletin affected the protein expression of AR/AR-V7. We were pleasantly surprised to find that although nobiletin had no significant effect on the protein level of the full-length AR (AR-FL), it downregulated AR-V7 in a dose- and time-dependent manner (Fig. 3A, B). Then immunofluorescence assay was performed to further verify the effect of nobiletin in downregulating the AR-V7 expression in 22Rv1 cells (Fig. 3C, D).

Next, we wondered how nobiletin downregulates AR-V7, such as by inhibiting AR-V7 protein synthesis or promoting its degradation. On one hand, real-time quantitative PCR assay was performed to detect the changes of AR-V7 mRNA in 22Rv1 cells treated with various concentrations of nobiletin, but the results showed that nobiletin did not downregulate AR-V7 mRNA levels (Fig. 3E), although nobiletin of 10–20 µM had caused a significant reduction in AR-V7 protein levels (Fig. 3A–D). On the other hand, cycloheximide (CHX), an inhibitor of eukaryotic translation [30], was used to test whether nobiletin accelerated the attenuation of AR-V7 protein levels in 22Rv1 cells. Not surprisingly, western blot assay showed that nobiletin destroyed the stability of AR-V7 protein (Fig. 3F, G). In addition, it is more noteworthy that this effect of nobiletin could be significantly reversed by bortezomib (BTZ), a 20 S proteasome inhibitor (Fig. 3H, I).

Furthermore, Co-IP assay was used to confirm that nobiletin instigated the K48-linked ubiquitinated form of AR-V7 (Fig. 3J, K). Formation of the K48-polyubiquitin chains on a substrate has been recognized as a key indicator of proteasome-mediated degradation of protein [31, 32]. These results demonstrate that nobiletin selectively promotes the proteasome-mediated AR-V7 degradation by sponsoring the K48-linked ubiquitination of AR-V7 protein in CRPC cells.

### Nobiletin prevents the interactions of AR-V7 with USP14 and USP22

In our previous research, the GRP78-AR-V7-SIAH2 axis has been reported in detail as the degradation pathway of AR-V7 [23]. Here we tried to explore whether there are new pathway or undiscovered regulatory factors for the degradation mechanism of AR-V7.

**Fig. 4:**
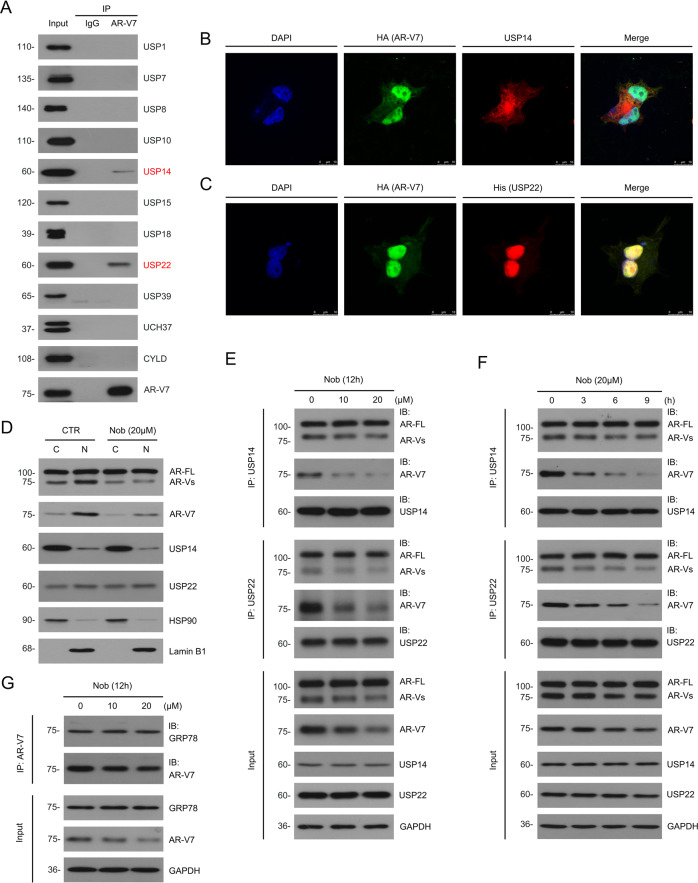
Nobiletin prevented the interactions of AR-V7 with USP14 and USP22. **A** Co-IP assays using AR-V7 antibody and immunoblot to indicated deubiquitinases in 22Rv1 cells. **B** Immunofluorescence assays using HA and USP14 antibodies in 22Rv1 cells transfected with HA-AR-V7 plasmids for 48 h. Scale bars represent 10 μm in shown images. **C** Immunofluorescence assays using HA and His antibodies in 22Rv1 cells co-transfected with His-USP22 and HA-AR-V7 plasmids for 48 h. Scale bars represent 10 μm in shown images. **D** Western blot analysis of AR, AR-V7 USP14, and USP22 protein level in cytoplasmic and nuclear of 22Rv1 cells treated with nobiletin for 24 h. HSP90 and Lamin B1 were used as cytoplasmic and nuclear internal controls, respectively. **E** Co-IP assay was performed to determine the interaction of USP14/USP22 and AR/AR-V7 in 22Rv1 cells exposed to nobiletin for 12 h. **F** Co-IP assay was performed to determine the interaction of USP14/USP22 and AR/AR-V7 in 22Rv1 cells exposed to nobiletin (20 μM) for different lengths of time. **G** Co-IP assay was performed to determine the interaction of GRP78 and AR-V7 in 22Rv1 cells exposed to nobiletin for 12 h.

Deubiquitylating enzymes (DUBs) mediate the reverse process of protein ubiquitination and is of great significance in the regulation of oncoprotein stability in various cancer cells [33–35]. To identify the potential DUBs of AR-V7, we first used the Co-IP assay to explore DUBs that could interact with AR-V7 even potentially regulate expression or function of AR-V7 in 22Rv1 cells. Through the screening of 11 DUBs, we showed that USP14 and USP22 interacted with AR-V7 (Fig. 4A). It should be noted that USP14 can interact with AR/AR-V7 and stabilize their protein levels by exerting the deubiquitination enzyme activity [24, 36], but the interaction between USP22 and AR-V7 is reported in the current research.

Next, to further explore the subcellular locations of the interactions between these proteins, we showed that the subcellular locations of USP14 and AR-V7 interaction were mainly in the cytoplasm, and small amount in the nucleus through immunofluorescence colocalization assay (Fig. 4B), and the subcellular locations of USP22 and AR-V7 interaction were mainly in the nucleus, and small amount in the cytoplasm (Fig. 4C). Then, we performed western blot assay to verify this phenomenon after nuclear and cytoplasmic separation. The results were highly consistent with immunofluorescence assay. In addition, the nuclear and cytoplasmic AR-V7 protein levels were downregulated by nobiletin, but the protein levels and subcellular distributions of USP14/USP22 were not affected (Fig. 4D).

To address whether USP14 and USP22 is critical in nobiletin-induced selective degradation of AR-V7, 22Rv1 cells were treated with nobiletin in the concentration gradient and time gradient as shown in the figure. Next, Co-IP and western blot experiments were performed to detect the interactions of AR-V7 with USP14 and USP22, respectively. We were pleasantly surprised to find that nobiletin notably prevented the interaction not only between USP14 and AR-V7, but also between USP22 and AR-V7, without affecting the levels of USP14 and USP22 (Fig. 4E, F). In addition, the interaction of USP22 and AR (including AR-FL and AR-Vs) was also confirmed. However, it is worth noting that nobiletin had no effect on the interactions between AR-FL and USP14/USP22, which may explain why nobiletin has a certain selectivity in inducing the degradation of AR-V7.

Our previous study has been demonstrated that rutaecarpinecan selectively trigger the GRP78-dependent AR-V7 degradation [23]. Hence, Co-IP assay was conducted to verify whether nobiletin also caused similar effects. Unlike rutaecarpine, nobiletin failed to alter the interaction of AR-V7 and GRP78 (Fig. 4G).

In summary, in addition to the reported USP14, USP22 was identified as another DUB that can interact with AR-V7. More importantly, their interactions were notably suppressed by nobiletin, which also propelled us to further explore the role of USP22 in the degradation pathway of AR-V7.

### USP14 and USP22 co-mediate the nobiletin-induced selective degradation of AR-V7 and suppression of CRPC cells

**Fig. 5:**
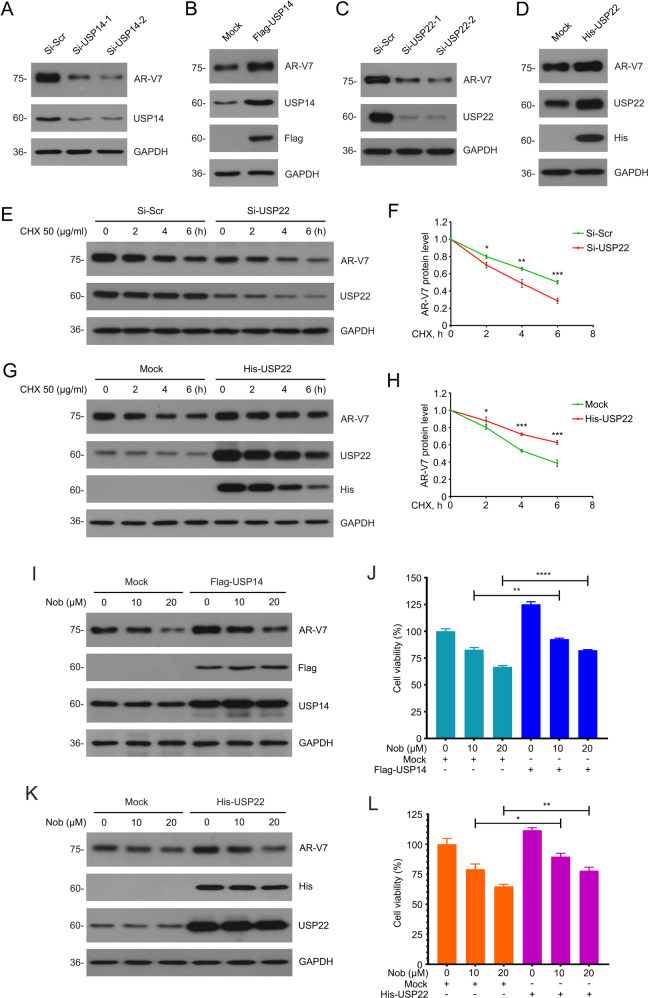
USP14 and USP22 co-mediated the induction of AR-V7 degradation and suppression of CRPC induced by nobiletin. **A** Western blot of AR-V7 and USP14 in 22Rv1 cells transfected with two pairs of USP14 or control siRNAs for 48 h. **B** Western blot of AR-V7, Flag, and USP14 in 22Rv1 cells transfected with Flag-USP14 plasmids or control plasmids for 48 h. **C** Western blot of AR-V7 and USP22 in 22Rv1 cells transfected with two pairs of USP22 or control siRNAs for 48 h. **D** Western blot of AR-V7, His, and USP22 in 22Rv1 cells transfected with His-USP22 plasmids or control plasmids for 48 h. **E** Western blot of AR and AR-V7 protein level in 22Rv1 cells transfected with USP22 siRNAs or scramble siRNAs for 48 h and then exposed to CHX. **F** Quantitative data of **E** are shown. Mean ± SD (*n* = 3). **G** Western blot of AR and AR-V7 protein level in 22Rv1 cells transfected with His-USP22 plasmids or control plasmids for 48 h and then exposed to CHX. **H** Quantitative data of **G** are shown. Mean ± SD (*n* = 3). **I** Western blot of AR-V7, Flag, and USP14 protein level in 22Rv1 cells transfected with Flag-USP14 plasmids or control plasmids for 48 h and then exposed to nobiletin for 24 h. **J** CCK8 assays were performed in 22Rv1 cells transfected with Flag-USP14 plasmids or control plasmids for 24 h and then exposed to nobiletin for 48 h. Mean ± SD (*n* = 3). **K** Western blot of AR-V7, His, and USP22 protein level in 22Rv1 cells transfected with His-USP22 plasmids or control plasmids for 48 h and then exposed to nobiletin for 24 h. **L** CCK8 assays were performed in 22Rv1 cells transfected with His-USP22 plasmids or control plasmids for 24 h and then exposed to nobiletin for 48 h. Mean ± SD (*n* = 3).

First, different siRNAs of USP14/USP22 and Flag/His-Tagged plasmid of USP14/USP22 were applied to evaluate the effect of USP14/USP22 on AR-V7 protein levels. To our expectation, the results showed that the knockdown of USP22 decreased AR-V7 expression. And correspondingly, overexpression of USP22 stabilized AR-V7 expression in a manner similar to USP14 (Fig. 5A–D), demonstrating that USP22 may also be involved in stabilizing the protein level of AR-V7, similar to USP14.

In fact, the regulation of AR expression and function by USP22 in prostate cancer has been reported [37], but whether AR-V7 could be regulated by USP22 is elusive. Our current research focuses on the regulation of AR-V7 protein stability mediated by USP22. Furthermore, CHX-tracking assay showed that USP22 depletion significantly decreased the half-life of AR-V7. In reverse, overexpression of USP22 slowed down the attenuation of AR-V7 (Fig. 5E–H). Therefore, we speculate that nobiletin can simultaneously inhibit the protective effects of USP14 and USP22 on AR-V7 protein levels, thereby suppressing the growth of CRPC.

To further verify our hypothesis, a series of reversal experiments were performed. Western blot and CCK8 assays showed that the overexpression of USP14/USP22 not only reversed the downregulation of AR-V7 level by nobiletin to a certain extent, but also partly weakened nobiletin-induced inhibition of the CRPC cell viability (Fig. 5I–L). These findings suggest that USP14/USP22 are key players in the nobiletin-induced selective degradation of AR-V7 and even the proliferative suppression of CRPC cells.

### Nobiletin enhances the sensitivity of CRPC to enzalutamide

**Fig. 6:**
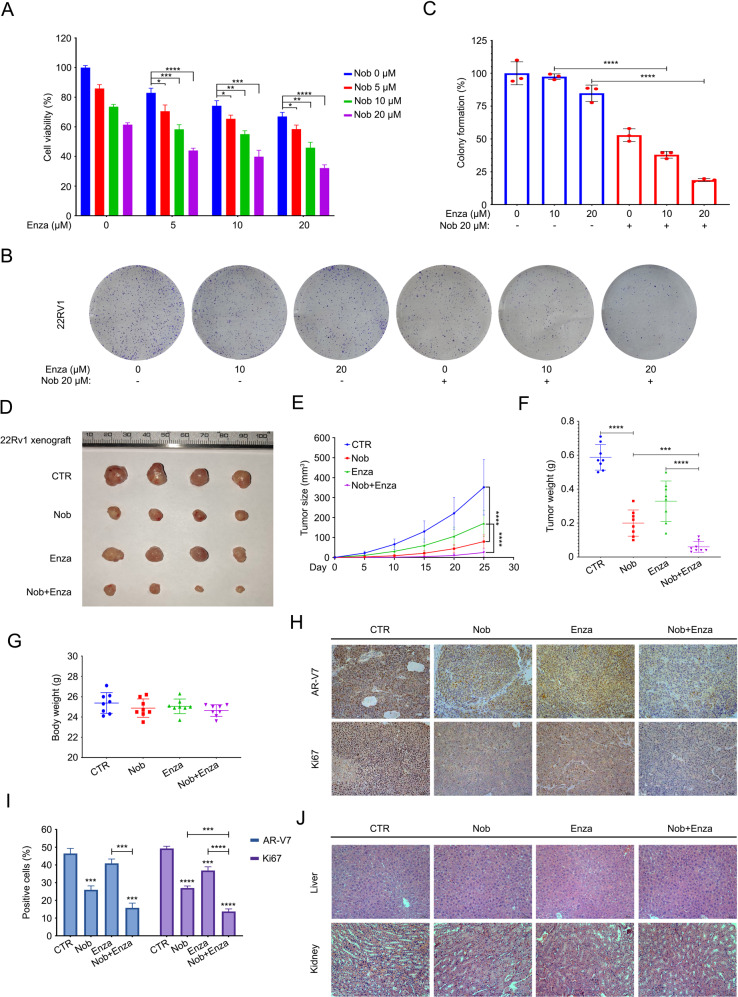
Nobiletin enhanced the sensitivity of CRPC to enzalutamide. **A** CCK8 assay was performed in 22Rv1 cells treated with enzalutamide in the presence or absence nobiletin for 48 h. Mean ± SD (*n* = 3). **B** Colony formation assay of 22Rv1 cells exposed to enzalutamide with or without nobiletin for 2 weeks. **C** Quantitative data of **B** are shown. Mean ± SD (*n* = 3). **D** Xenograft images are shown. 22Rv1 xenografts treated with drug solvent, 40 mg/kg/2d (i.p.) of nobiletin, 25 mg/kg/2d (p.o.) of enzalutamide and combination of nobiletin and enzalutamide for 25 days. **E** Tumor sizes are shown. Mean ± SD (*n* = 10). **F** Tumor weight are shown. Mean ± SD (*n* = 10). **G** Body weight of nude mice are shown. Mean ± SD (*n* = 10). **H** IHC assay of AR-V7 and Ki67. Representative images in per group are shown at 200*. **I** Quantitative data of **H** are shown. Mean ± SD (*n* = 3). **J** H&E staining assay was performed in liver and kidney tissue of nude mice. Representative images in per group are shown at 200*.

AR-V7 is a driver to boost the occurrence and development of CRPC [5, 38, 39]. After demonstrating the effect of nobiletin-induced downregulation of AR-V7 and its mechanism. We next wondered whether nobiletin can enhance the sensitivity of CRPC to antiandrogen therapy due to its selective degradation of AR-V7. First, CCK8 and the colony formation experiments showed that nobiletin significantly strengthened the inhibitory effect of enzalutamide (a second-generation androgen receptor signaling inhibitor) on the cell viability and colony formation ability of 22Rv1 cells in vitro (Fig. 6A–C). To further define whether nobiletin could achieve similar effects in vivo, we established 22Rv1 xenografts under the skin of nude mice and then randomly divided into four groups. Mice were treated with nobiletin, enzalutamide, alone or their combination. We found that the growth of xenografts in the nobiletin treatment group was inhibited compared with the control group. In addition, the difference in xenografts growth between the combination treatment group and the enzalutamide treatment group was also statistically significant (Fig. 6D–F). These findings suggest that nobiletin enhanced the sensitivity of CRPC to enzalutamide in vitro and in vivo.

Immunohistochemistry assay further verified the effect of nobiletin in the downregulation of AR-V7 and Ki67 in vivo (Fig. 6H, I). Moreover, there was no significant difference in the body weight of the four groups (Fig. 6G). The tissue damage in liver and kidney cannot be observed in mice (Fig. 6J), indicating that the side effects of nobiletin/enzalutamide alone or in combination were low.

### Nobiletin suppresses the growth of CRPC cells depending on the status of AR-V7

**Fig. 7:**
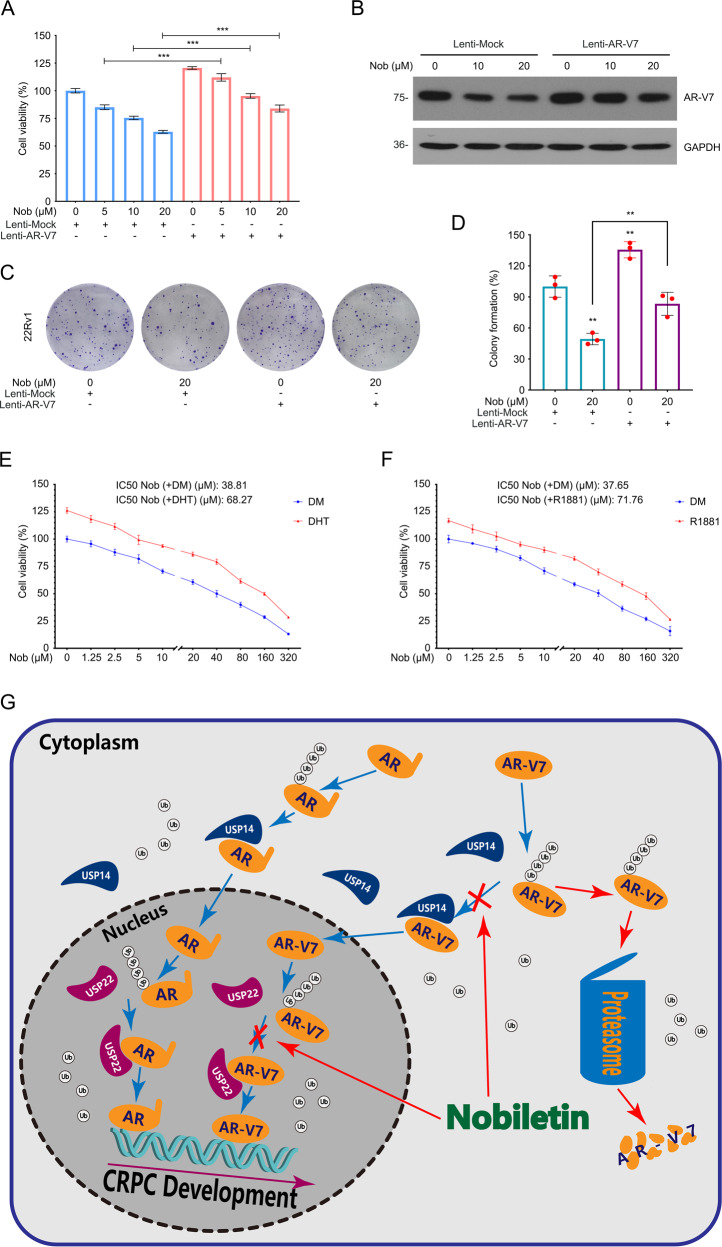
Nobiletin induced AR-V7-dependent inhibition of proliferation in PC. **A** CCK8 analysis of 22Rv1 cells transfected with lentiviruses containing AR-V7 plasmids or control plasmids for a week and then exposed to nobiletin for 48 h. Mean ± SD (*n* = 3). **B** Immunoblot of AR-V7 in 22Rv1 cells transfected with lentiviruses containing AR-V7 plasmids or control plasmids for a week and then exposed to nobiletin for 24 h. **C** Colony formation assay of 22Rv1 cells transfected with lentiviruses containing AR-V7 plasmids or control plasmids for a week and then exposed to nobiletin for 2 weeks. **D** Quantitative data of **C** are shown. Mean ± SD (*n* = 3). **E**, **F** CCK8 analysis of 22Rv1 cells pretreated with or without DHT (10 nM) and R1881(10 nM) for 48 h and then exposed to nobiletin for 48 h. Mean ± SD (*n* = 3). **G** A proposed model of nobiletin-induced anti-CRPC activity.

To further explore the relationship between the antitumor effect of nobiletin and its promotion of AR-V7 protein degradation, an AR-V7-overexpressing 22Rv1 cell line that stably expressing AR-V7 was constructed by using lentiviruses containing AR-V7 plasmids. CCK8, colony formation, and immunoblot assays were performed in these cells exposed to nobiletin. The results showed that nobiletin-induced inhibition of AR-V7, cell viability, and colony formation ability can be significantly reversed by overexpressing AR-V7 (Fig. 7A–D), suggesting that the nobiletin-induced suppression of CRPC depends on AR-V7 status.

In addition, we also would like to verify whether the inhibitory effect of nobiletin on CRPC proliferation is different with or without androgen stimulation, although we deem that nobiletin does not pose a sufficient threat to full-length AR that is activated by androgens to promote CRPC progression. We pretreated 22Rv1 cells with or without Dihydrotestosterone (DHT) and the synthetic androgen Metribolone (R1881), then performed CCK8 analysis for quantitating the inhibitory effect of nobiletin on 22Rv1 cell viability. The results showed that androgen stimulation weakened the suppression of CRPC by nobiletin to a certain extent (Fig. 7E, F), which may further imply the necessity of nobiletin combined with antiandrogen drugs.

## Discussion

Natural products have become one of the historically rich sources of drug research and development. A large number of natural compounds were proposed to display unexpected anticancer effects and low side effects [23, 40–42], implicating that the researches on the anticancer mechanism of natural products are very promising.

It has been reported that nobiletin inhibits the growth of prostate cancer cells by suppressing TLR4/TRIF/IRF3, TLR9/IRF7 [43] and AKT [44] signaling pathways. In our current study, we observed that nobiletin caused the inhibition of the cell viability of prostate cancer cells, especially AR/AR-V7-positive cells. Next, additional proliferation experiments further verified that nobiletin more significantly induced the growth inhibition of AR/AR-V7-positive prostate cancer cells. Similarly, DeveciOzkan A, et al. found that LNCaP (an AR/AR-V7-positive prostate cancer cell line) cells are more sensitive to nobiletin than PC3 cells and speculated that the difference may be related to AR. However, the role of AR/AR-V7 in the nobiletin-induced PC suppression has not been explored [43]. In our observations, overexpression of AR-V7 could rescue the inhibition of cell viability and colony forming ability caused by nobiletin, which further demonstrated that nobiletin suppressed CRPC proliferation depends on AR-V7. Furthermore, we did not observe significant apoptosis induced by nobiletin, which is inconsistent with the study of Tang M, et al. [45]. It may be caused by the different concentration and time of nobiletin treatment because high concentrations (65, 100, and 130 μM) of nobiletin and 72 h were selected in their study. More importantly, we further showed that nobiletin not only had a satisfactory anticancer effect, but also enhanced the sensitivity of CRPC to enzalutamide with low toxic and side effects through in vivo and in vitro experiments.

In order to explore why AR/AR-V7-positive prostate cancer cells are more sensitive to nobiletin, western blot and immunofluorescence assays were performed. We showed that nobiletin at relatively low concentrations (10 and 20 μM) can significantly decrease the expression levels of AR-V7 in CRPC cells, but has no obvious impact on AR-FL. In addition, the real-time quantitative PCR assay help us rule out the possibility of nobiletin on downregulating the transcription level of AR-V7. Furthermore, the CHX-tracking and BTZ-protein degradation reversal experiments consistently confirmed that nobiletin induced AR-V7 protein degradation through the ubiquitin-proteasome pathway. More directly, we found that nobiletin elevated the level of K48-linked polyubiquitination of AR-V7 via Co-IP assay.

DUBs, a series of catalytic enzymes, mediate the ubiquitination progression through removing the ubiquitin or polyubiquitin chain from their specific substrate. So it is essential for regulating the stability and function of the substrate protein [46]. In our previous researches, we have been explored the effect of USP14 on the growth of prostate cancer [24] and breast cancer [28] cells via regulating the stability of AR. Here, we tried to identify potential DUBs that interact with AR-V7 for increasing our knowledge on the molecular mechanism of AR-V7 degradation. We confirmed the endogenous interaction between AR-V7 and USP22. USP22 is initially identified as acomponent of human Spt–Ada–Gcn5 acetyltransferase (SAGA) complex to modulate gene transcription via mono-deubiquitylation of histones H2A and H2B in the nucleus [47, 48]. In prostate cancer, it has been reported that USP22 drives carcinogenic effects by regulating cell proliferation and DNA repair [49]. In addition, another study has identified that USP22 is a major effector of AR levels, AR output, AR-MYC coordination, and the transition to CRPC to drive a lethal cancer progression [37]. In their study, USP22 regulates proteasome-dependent endogenous AR degradation in PC cells. Our CHX-tracking assay confirmed that USP22 promoted the stability of AR-V7 protein. Immunofluorescence and western blot experiments showed that these molecular events mainly occurred in the nucleus.

We also confirmed that nobiletin can significantly decrease the interactions between AR-V7 and USP14/USP22 in a time and concentration-dependent manner, but did not affect the interactions between AR-FL and USP14/USP22, which may explain why nobiletin exhibits a certain selectivity in inducing the degradation of AR-V7. Furthermore, the overexpression of USP14/USP22 reversed the degradation of AR-V7 induced by nobiletin to a certain extent, and partly recovered the viability of CRPC cells. Our previous research also revealed that the GRP78-AR-V7-SIAH2 protein complex mediates AR-V7 degradation [23]. We therefore wondered whether nobiletin has a similar effect on the interaction between GRP78 and AR-V7. Unlike rutaecarpine, nobiletin failed to alter the GRP78-mediated degradation of AR-V7.

In conclusion, this research not only demonstrates the reason why nobiletin suppressed the growth process of CRPC through the selective degradation of AR-V7, but also enriches our understanding of the degradation mechanism of AR-V7 and provides an efficient treatment target to overcome CRPC via targeting the interaction between AR-V7 and USP14/USP22 (Fig. 7G).

## Supplementary information

Supplementary Data

## Data Availability

All the data and material supporting the conclusions were included in the main paper.
