# Supplementary material for: Selective degradation of AR-V7 to overcome castration resistance of prostate cancer
Source: Cell Death Dis. 2021 Sep 21;12(10):857. doi: 10.1038/s41419-021-04162-0 (PMC8455663; doi:10.1038/s41419-021-04162-0)
Supplement: Supplementary file 1 — Supplementary Data [file 41419_2021_4162_MOESM1_ESM.docx]

**Table S1.Information of antibodies used in the study.**

| **Antibodies** | **Manufacturer** | **Catalog number** | **Application** | **Dilution** |
| --- | --- | --- | --- | --- |
| anti-CDK2 | Cell Signaling Technology | #2546 | WB | 1:1000 |
| anti-CDK4 | Cell Signaling Technology | #12790 | WB | 1:1000 |
| anti-CDK6 | Cell Signaling Technology | #13331 | WB | 1:1000 |
| anti-cyclinD1 | Cell Signaling Technology | #2978 | WB | 1:1000 |
| anti-p15 | Cell Signaling Technology | #4822 | WB | 1:1000 |
| anti-p21 | Cell Signaling Technology | #2947 | WB | 1:1000 |
| anti-p27 | Cell Signaling Technology | #3686 | WB | 1:1000 |
| anti-GAPDH | Cell Signaling Technology | #5174 | WB | 1:1000 |
| anti-AR | Cell Signaling Technology | #5153 | WB | 1:1000 |
| anti-AR-V7 | Cell Signaling Technology | #19672 | WB | 1:1000 |
|  |  |  | IP | 1:50 |
| K48-linkage Specific Polyubiquitin | Cell Signaling Technology | #12805 | WB | 1:1000 |
| anti-IgG | Cell Signaling Technology | #3900 | IP | 1:50 |
| anti-USP1 | Cell Signaling Technology | #8033 | WB | 1:1000 |
| anti-USP7 | Cell Signaling Technology | #4833 | WB | 1:1000 |
| anti-USP8 | Cell Signaling Technology | #11832 | WB | 1:1000 |
| anti-USP10 | Cell Signaling Technology | #8501 | WB | 1:1000 |
| anti-USP14 | Cell Signaling Technology | #11931 | WB | 1:1000 |
|  |  |  | IP | 1:50 |
|  |  |  | IF | 1:200 |
| anti-USP15 | Cell Signaling Technology | #66310 | WB | 1:1000 |
| anti-USP18 | Cell Signaling Technology | #4813 | WB | 1:1000 |
| anti-USP22 | Abcam | #ab195289 | WB | 1:1000 |
|  |  |  | IP | 1:50 |
| anti-USP39 | Abcam | #ab131244 | WB | 1:1000 |
| anti-UCHL5/UCH37 | Abcam | #ab236002 | WB | 1:1000 |
| anti-CYLD | Cell Signaling Technology | #8462 | WB | 1:1000 |
| anti-HSP90 | Cell Signaling Technology | #4877 | WB | 1:1000 |
| anti-Lamin B1 | Cell Signaling Technology | #13435 | WB | 1:1000 |
| anti-GRP78 | Abcam | #ab21685 | WB | 1:1000 |
| anti-His-Tag | Cell Signaling Technology | #12698 | WB | 1:1000 |
|  |  |  | IP | 1:50 |
| anti-HA-tag | Cell Signaling Technology | #3724 | WB | 1:1000 |
|  |  |  | IF | 1:200 |
| anti-HA-tag | Cell Signaling Technology | #2367 | IF | 1:200 |
| anti-DYKDDDDK (Flag)-Tag | Cell Signaling Technology | #14793 | WB | 1:1000 |
| anti-rabbit IgG (HRP Conjugate) | Cell Signaling Technology | #7074 | WB | 1:5000 |
| anti-mouse IgG (HRP Conjugate) | Cell Signaling Technology | #7076 | WB | 1:5000 |
| Mouse Anti-rabbit IgG (HRP Conjugate) | Cell Signaling Technology | #5127 | WB | 1:2000 |
| anti-AR-V7 | Abcam | #ab198394 | IHC | 1:200 |
| anti-Ki67 | Abcam | #ab15580 | IHC | 1:200 |

**Table S2.Information of chemicals used in the study.**

| **Chemicals** | **Manufacturer** | **Catalog number** |
| --- | --- | --- |
| Nobiletin | Selleck | #S2333 |
| Bortezomib | Selleck | #S1013 |
| MG132 | Selleck | #S2619 |
| Enzalutamide | Selleck | #S1250 |
| Dihydrotestosterone (DHT) | Selleck | #S4757 |
| Cycloheximide | Cell Signaling Technology | #2112 |
| R1881 | AbMole | #M8128 |
